# Production Significance of Bovine Respiratory Disease Lesions in Slaughtered Beef Cattle

**DOI:** 10.3390/ani10101770

**Published:** 2020-09-30

**Authors:** Miguel Fernández, María del Carmen Ferreras, Francisco Javier Giráldez, Julio Benavides, Valentín Pérez

**Affiliations:** 1 Departamento de Sanidad Animal, Facultad de Veterinaria, Universidad de León, E-24071 León, Spain; mcfere@unileon.es (M.d.C.F.); vperp@unileon.es (V.P.); 2 Instituto de Ganadería de Montaña (IGM), CSIC-Universidad de León, E-24346 León, Spain; j.giraldez@eae.csic.es (F.J.G.); j.benavides@eae.csic.es (J.B.)

**Keywords:** pneumonia, respiratory, lung, bovine, impact, disease

## Abstract

**Simple Summary:**

Respiratory diseases are a common health and economic problem in beef cattle production. Bovine respiratory syndrome, by which these processes are known, needs to be studied in different aspects. Therefore, this study has tried to understand the impact on the production of this disease through the study of the post-mortem lesions observed in clinically healthy beef animals and its associated factors for the management system, age and gender. It was found that there is a high percentage of subclinical pneumonia, both chronic and acute fibrinous, with different grade expanse. The animals that showed lesions experienced a lower average carcass weight than those without lesions. Furthermore, the mixed and extensive production system, as well as young animals, are associated with a greater probability of presenting pneumonic lesions. The bacterial agents were only identified in the acute fibrinous pneumonia. This study reflects the high prevalence of animals arriving at the slaughterhouse that suffer or have suffered respiratory disease and can serve as herd monitoring to adopt the necessary health measures to avoid economic losses.

**Abstract:**

Bovine respiratory disease (BRD) is still a serious concern in feedlots, where it exerts a negative effect on farm productivity. There is a shortage of studies focused on the evaluation of BRD-associated lesions at the slaughterhouse in clinically healthy animals. The objective of this work was to investigate the prevalence and type of subclinical pneumonic lesions in slaughtered beef cattle, according to the age range and management system, and its impact on carcass weight. A total of 1101 beef cattle intended for human consumption were examined at slaughter. Information on age, sex, management system and carcass weight was recorded. The presence and type of pneumonia were evaluated according to gross and microscopic findings and etiological agents by PCR. Lung pneumonic lesions appeared in 17.9% of animals and were predominant among veal calves. According to the type, chronic catarrhal pneumonia prevailed in the majority of animals, and mixed and extensively reared cattle were more likely to suffer acute fibrinous pneumonia. The presence of pneumonic lesions was associated with a significant decrease in carcass weight that had more of an impact in veal male calves coming from intensive systems. Bacterial infections were the predominant infectious agent and the only cause of acute fibrinous pneumonia, while viruses were infrequent and only found in lesions with chronic catarrhal pneumonia. This study shows the importance of BRD in beef feedlots upon production values and points out the feasibility of slaughterhouse assessment of pneumonia as a method for the evaluation of BRD significance.

## 1. Introduction

Bovine respiratory disease (BRD) is regarded as the most common illness during the fattening period of cattle in Europe and North America [1,2,3] and the most important cause of economic burden to the feedlot industry due to its high morbidity and mortality [4,5]. It has been estimated that it is responsible for 69% of the total casualties in a feedlot [6,7]. BRD’s economic costs are primarily due to metaphylactic and therapeutic use of antibiotics, and the loss of weight of affected animals [1,8,9]. The negative effect of BRD on carcass composition and quality traits also has been demonstrated [9,10]. Costs can be up to 200 USD per calf [5] and account for 7% of total production costs in North America, amounting to approximately 1 billion USD [1,2,11].

Pneumonia is the principal feature of the BRD complex. Clinical signs include depressed demeanor, loss of appetite, increment of cough frequency, nasal discharge accompanied by febrile episodes and respiratory insufficiency [12]. Pneumonia associated with BRD is considered a multifactorial disease resulting from interactions between infectious agents, such as bacteria or viruses [4,13], and extrinsic factors such as stress caused by deficient climatic conditions, dehorning, weaning, transportation or immunosuppression periods caused by other viral agents [14,15,16].

In a study performed in Canadian feedlots, the most common infectious agents of bacterial etiology found in pneumonic lungs by polymerase chain reaction (PCR) were *Mycoplasma bovis* (36%), *Mannheimia haemolytica* (27%), *Pasteurella multocida* (19%) and bovine viral diarrhea virus (BVD) (35%), bovine respiratory syncytial virus (BSRV) (9%), bovine herpesvirus serotype 1 (BHV-1) (6%) and parainfluenza virus serotype 3 (PI-3) (3%) regarding viruses [6]. Mixed infections are frequently identified in BRD pneumonia, and the etiology of the infection is highly related to the type of pulmonary lesions and duration [6,8,17,18].

Assessment of the prevalence of pneumonic lesions at the slaughterhouse can be a good indicator of the relevance of BRD in beef cattle [3,19,20]. However, there is a shortage of studies that assess the relationship between the presence of lesions within the individual with productive factors that can influence them at the end-point of fattening efficiency. There are scant works in relation to BRD in Europe [19,21] and particularly in Spain, where, despite the importance of the beef industry, which comprises approximately 2 million individuals [22], only a few descriptive data of some local administration are available [23].

The objectives of this research were to investigate the prevalence of pneumonia in clinically healthy veal calves and yearlings based on the examination of lesions related to BRD at the slaughterhouse and to determine the effect of production system, age, sex and the impact of subclinical pneumonia on weight. In addition, this study aimed to classify BRD-related pneumonia according to the type of lesion and also to identify, via PCR, the infectious causes associated with the different types.

## 2. Material and Methods

Experimental animals were not used in this work. An observational study has been performed with the data and with post mortem samples that are routinely collected in a slaughterhouse.

### 2.1. Animals

A total of 1101 beef-breed cattle, intended for consumption and without previous respiratory clinical signs, were post-mortem examined at an authorized slaughterhouse under the European Union regulations. All the animals came from feedlots located in Castile and León (Northwestern Spain). The visits at the slaughterhouse were carried out during four consecutive months (from September to December 2017). The animals participating in the study were subjected to the strictest traceability measures, and individual data were also provided by the feedlot administration. Upon arrival at the slaughterhouse, individual identification was verified. Animals were examined at arrival by official veterinarians in accordance with all the veterinary criteria scheduled for the antemortem examination included in Spanish legislation. The absence of clinical symptoms was established according to feedlot records and the antemortem veterinary official examination.

All animals under the study were categorized according to the most common production systems for beef cattle in Spain: intensively managed (animals were kept indoors with controlled feeding and no access to pasture), extensive management (animals remained in the pasture all the time) and mixed system (animals were on pasture for variable periods of their feeding and grazing seasons, but indoors the rest of the time). In every case, data related to the management system, gender and age type (veal calves up to 12 months old; yearlings between 12 and 24 months old) were recorded (Table 1). For each animal, carcass weight was also measured after evisceration in the slaughterhouse. Detailed information on animal identification, weight and farming type was provided by the slaughterhouse veterinarians and the Livestock Department of the Castile and León regional government.

### 2.2. Sampling

Samples from the affected areas on every lung showing gross lesions were collected according to standardized procedures at the slaughterhouse and good laboratory practices to avoid contamination and ensure data quality. Lung samples were taken and stored in individual sterile freezer bags at −80 °C until analysis by PCR assay. Samples from the same areas were fixed in 10% buffered formalin for 48 h and dehydrated through a graded alcohol series before being embedded in paraffin wax. Cut sections (3.5 μm) were obtained from each sample and stained with hematoxylin and eosin (HE) for histological examination.

### 2.3. Macroscopic and Microscopic Inspection of the Lungs

Initial assessment of the lungs comprised the evaluation of the distribution and location of macroscopic changes consistent with pneumonia such as variations in color (from red to grey), presence of consolidation areas, or exudate. Examination was performed under the agreement of two pathologists (MF and MCF) in accordance with the guidelines and criteria for classification of bovine pneumonic lesions [24,25]. All the lungs with gross lesions, subjected to the same traceability as their carcass, were separated for more detailed examination, including cross-sectioning to ensure the optimal macroscopic evaluation and data control. Macroscopic lesions were post-hoc classified into two different types: chronic catarrhal, characterized by well-demarcated, purple to grey in color, cranioventral consolidated areas, with firm texture and no increase of volume; and acute fibrinous pneumonia, characterized by the presence of well-demarcated solid and swollen cranioventral areas with evident vascular reaction, such as congestion or fibrin deposition over the pleura of the affected parts. To classify the lesion expanse, a subjective assessment using a previously established scoring system was performed [2]. Grade 1 or minor pneumonia was assigned to those cases in which the affected area was equal to or less than approximately 10% of the lung and grade 2 or extent pulmonary disease, in which the lesion concerned more than 10% of the pulmonary parenchyma.

Sections were microscopically analyzed and evaluated rigorously and independently by MF and MCF or, in case of disagreement, by VP. Histologically, lesions were post-hoc classified into two main types: chronic catarrhal and acute fibrinous pneumonia. The guidelines and microscopic findings already described and proposed for characterization of bovine pneumonic lesion [25,26] were employed.

### 2.4. Etiological Identification by PCR

This technique was only performed on a representative number of samples, chosen according and proportionally to the different type of lesion found. Etiological identification by RT-PCR was performed on a total of 50 randomly chosen samples, representative of each type of pneumonia: 40 from chronic catarrhal bronchopneumonia lesions and the remaining 10 from the acute fibrinous pneumonia group.

DNA extraction was carried out under sterile conditions in a vertical laminar flow cabinet. Tissue (25 mg) from each sample was cut with disposable sterile blades into small pieces and put into sterile Eppendorf tubes. Thereafter, the DNA and RNA extraction was performed using the Speedtools Tissue DNA Extraction Kit^®^ (Biotools B&M Labs S.A, Madrid, Spain) and the Speedtools Total RNA Extraction kit^®^ (Biotools B&M Labs S.A, Madrid, Spain), respectively, according to the manufacturer’s instructions. Eluted DNA was stored in a sterile eppendorf at −20 °C and total RNA at −80 °C until analysis by real-time PCR (RT-PCR).

Etiological agents assessed were those most commonly reported in BRD cases [4,18]. RT-PCR amplification of genomic regions of *Mycoplasma bovis*, *Histophilus somni, Mannheimia haemolytica, Pasteurella multocida*, Bovine Herpesvirus type 1 (BHV-1), Bovine Viral Diarrhea Disease Virus (BVDV), Bovine Respiratory Syncytial Virus (BRSV) and Parainfluenza Virus type 3 (PI-3) was carried out using commercial kits according to the instructions of each manufacturer on a conventional thermocycler ABI 7500 Real-Time PCR System (Applied Biosystems^®^, Foster City, CA, USA) with the corresponding cycling parameters (Supplementary Table S1). Samples were processed in duplicate for each kit, all at once, with the same equipment and by the same person (MF) to assure the laboratory measurements and avoid variability.

### 2.5. Statistical Analysis

The inter-observer agreements for the histopathological classification of pneumonia and the expanse of lesions were calculated through the weighted *kappa* (wκ) and Cohen’s *kappa* (κ) statistics, respectively.

Several models to fit the logit of the odds (log-probability of the event/probability of the no event) of different events (pneumonia vs. healthy animals, acute fibrinous vs. chronic pneumonia or severe vs. mild lesions) were constructed and tested using the GLIMMIX procedure of SAS version 9.4 (SAS Institute Inc, Cary, NC, USA). The models included the system of production (intensive, mixed and extensive), animal type (veal vs. yearling) and sex (male vs. female) as fixed effects and the farm nested to the system as a random effect. When the severity of the lesion was evaluated, the type of pneumonia was also included as fixed effect in the model. When possible, models including double interactions were tested, and those models whose Pearson chi-square/DF value was nearest to 1 were selected, in order to avoid the effect of overdispersion on probability values. Random effect of the farm (system) was dropped from the model when its variance was zero. Adaptive quadrature of the Gauss–Hermite method was used for computing the maximum likelihood (true). Pearson chi-square values were 0.99, 0.96 and 0.99 for the final selected to predict the log odds of pneumonia, type of pneumonia (acute fibrinous) and severity of lesions (grade II), respectively.

Carcass weight and age-at-slaughter data were subjected to analysis of variance using the MIXED procedure of SAS. Health status, production system, type of animal and sex were included as fixed effects in the statistical model, farm nested to system being included as a random effect. Double and triple interactions were also included in the models and dropped when the *p*-value was greater than 0.20. In order to get as much information as possible, a second analysis was performed to study separately the effects of the type of pneumonia and severity of the lesion.

## 3. Results

### 3.1. Prevalence of Pneumonia

Lung lesions consistent with pneumonia were found in 198 out of the 1101 animals studied (17.9%). Regarding the age of the affected animals, 145 out of 753 (19.4%) within the veal group showed lesions, while in the yearling group, pneumonia was found in 53 out of 348 (15.3%) (Table 2 and Supplementary Table S2). The production system and type of animal were included in the predictive model for the prevalence of pneumonia. Overall, statistical analysis showed an association (*p* < 0.05) for subclinical pneumonia to occur in veal calves more frequently than in yearlings. The production system reflected a highly significant prevalence of pneumonia in veal calves raised in mixed (27.8%; *p* < 0.01) and extensive management (35.7%; *p* < 0.05) systems. The case of an animal to be classified as suffering pneumonia increased when animals came from extensive or mixed management systems and decreased with animal age (from veal to yearling) (Table 2).

### 3.2. Type of Pneumonia

Lesions consistent with pneumonia were classified into two groups according to the gross and histological findings: catarrhal chronic bronchopneumonia and acute fibrinous pneumonia.

**Figure 1:**
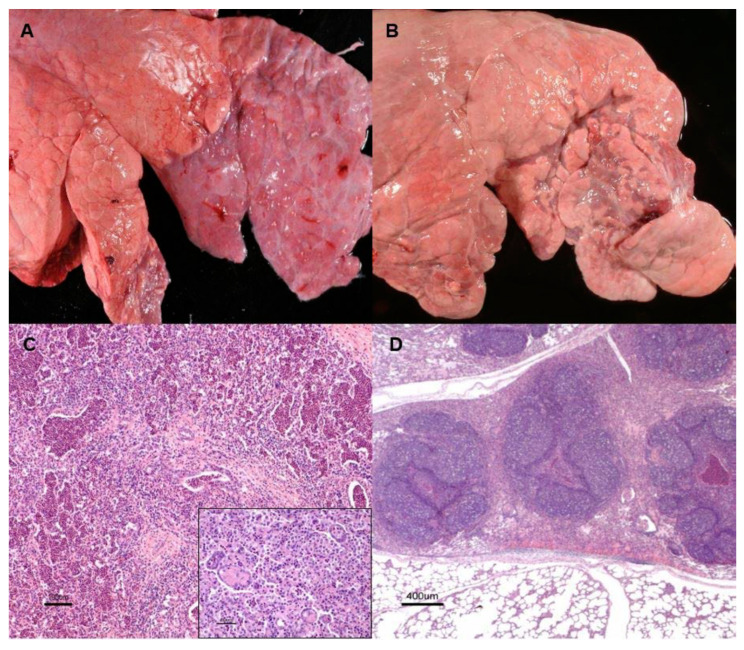
Chronic catarrhal bronchopneumonia. (**A**). Demarcated area of cranioventral lung consolidation with purple tonality, firm consistency and no increase of volume. Mucopurulent content and bronchiectasis were observed (inset). (**B**). Lesion showing recovery features such as coalescing small purple foci, consistent mainly with atelectasis surrounded with lobules with variable degrees of emphysema. (**C**). Haematoxylin-eosin staining (H-E). Histologically, neutrophils and, less in number, macrophages, occupy the bronchioalveolar lumen; foci of newly epithelization (inset) were observed. (**D**). Areas corresponding with atelectasis associated with occlusion of the bronchiolar lumen by inflammatory exudate and the peripheric lymphoid hyperplasia.

**Figure 2:**
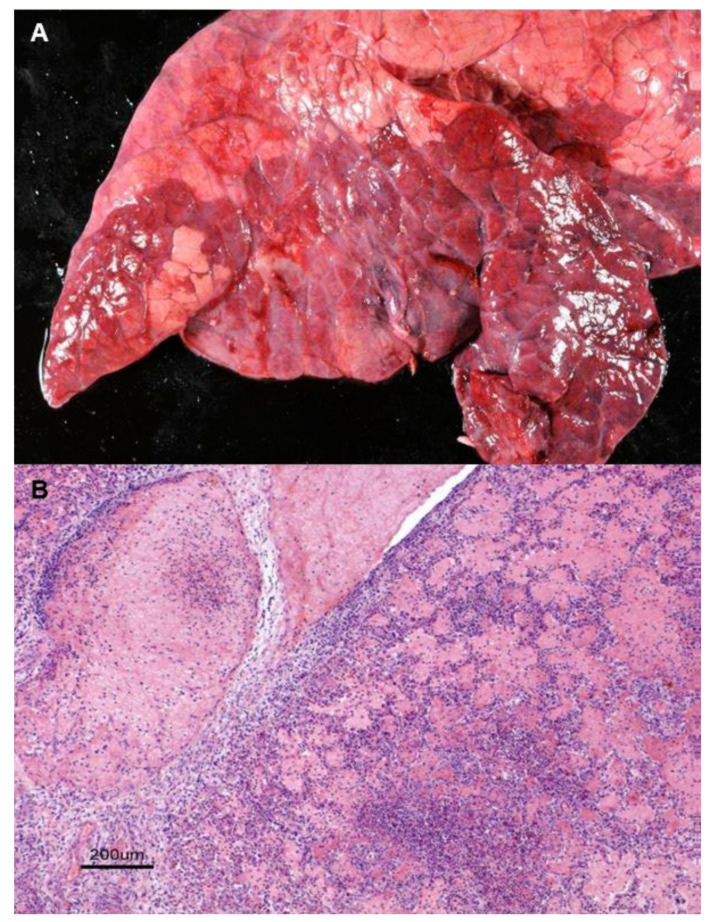
Acute fibrinous pneumonia. (**A**). Swollen, firm and well-demarcated cranioventral areas with vascular signs, accompanied by deposits of fibrin on the pleural surface. Haematoxylin-eosin staining (H-E). (**B**). Histologically, neutrophils, macrophages and large amounts of fibrin casts are present in the alveolar spaces with small foci of coagulative necrosis.

(a) *Catarrhal chronic bronchopneumonia* is characterized by well-demarcated, purple to grey in color and cranioventral-consolidated areas, with firm texture and no increase of volume (Figure 1A). In some cases, bands of obstructive atelectasis with a variable degree of alveolar emphysema and small pneumonic areas (Figure 1B) were the main lesions observed. On the cut section, affected zones were characterized by the presence of a variable amount of mucopurulent exudate with bronchiectasis and thickening of the bronchial wall. Histologically, the main finding was the presence of an inflammatory infiltrate formed by neutrophils and, to a lesser extent, macrophages in the bronchioalveolar lumina, newly made alveolar epithelization (Figure 1C) and areas with peribronchiolar lymphoid hyperplasia (Figure 1D).

(b) *Acute fibrinous pneumonia* is characterized by the presence of well-demarcated solid and swollen cranioventral areas with evident vascular reaction, such as congestion or fibrin deposition over the pleura of the affected parts (Figure 2A). Microscopically, fibrin casts were observed in the alveolar spaces together with an inflammatory exudate composed of neutrophils and macrophages and, occasionally, small foci of coagulative necrosis (Figure 2B).

Pathologist agreement was almost perfect according to the weighted kappa inter-observer analysis (wκ = 0.93).

Chronic catarrhal pneumonia was the most common type found in animals from all the management systems (87.4%) regardless of age or gender (*p* < 0.01). The probability that subclinical pneumonia was diagnosed as acute fibrinous pneumonia increased when the animal came from either semi-extensive (*p* < 0.05; Odds ratio (OR) > 2.86) or extensive (*p* < 0.01; OR > 5) production systems in comparison to those reared in intensive systems. However, no association was seen between the type of pneumonia, age of the animals or gender (Table 3).

### 3.3. Affected Area

Following the classification system previously proposed [2], animals with grade 1 lesions (<10% of parenchyma) were significantly more frequent (117 out of 198, 59.1%) than those with grade 2 (>10%) (81 animals, 40.9%) (*p* < 0.05). In this group, the lesion extension of the lesion never exceeded 30% of the lung parenchyma. Cattle showing grade 2 lesions were more commonly reared in mixed systems (*p* < 0.05). Additionally, no association could be established with the rest of the assessed parameters, such as type of pneumonia, gender or age (Supplementary Table S3). The interoperator kappa agreement to classify the expanse of the lesion was moderate (κ = 0.83).

### 3.4. Carcass Weight

Animals with pneumonic lesions showed a significant decrease (*p* < 0.05) in the average carcass weight compared to those without lesions. When considering the production system, there were significant differences (*p* < 0.05) in carcass weight between healthy animals and those with pneumonia when intensively managed. Slaughtered veal calves suffering from pneumonia tended to have a significantly lower carcass weight (*p* < 0.1) than those animals from the same group of age with no lung lesions, whereas in yearlings, no differences were apparent. (*p* > 0.05). The effect of pneumonia on carcass weight was sex-dependent (*p* < 0.001), and this effect was only significant in male animals (*p* < 0.05). It is noteworthy that the effect of farm nested to production system on carcass weight was not significant, but this factor has an influence on the weight when the age type and sex of the animals are considered (*p* < 0.0001) (Table 4).

No particular differences in carcass weight were found in relation to the type of pneumonia or lesion extent, with no differences between grade 1 and grade 2.

### 3.5. Age at Slaughter

In animals with pneumonia, the age at slaughter (fattening days) was significantly lower in the group of intensively reared beef (*p* < 0.05). However, in those coming from a mixed management system, fattening days tended to be higher than animals without lesions (*p* < 0.1).

In this sense, regarding gender, males with pneumonic lesions are prone to reach the slaughterhouse time point earlier than the healthy ones (*p* < 0.1) (Table 5).

According to the age group of animal, veal calves or yearlings, no differences were found (*p* > 0.05). No significant differences were found in the age at slaughter in relation to lesion severity or type of pneumonia (*p* > 0.05).

### 3.6. Prevalence of Etiological Agents

Etiological identification by RT-PCR was performed on a total of 50 randomly chosen samples, representative of each type of pneumonia: 40 from chronic catarrhal bronchopneumonia lesions and the remaining 10 from the acute fibrinous pneumonia group. The distribution of the cases was made proportionate to the total number of animals presenting each type of pneumonia. Forty-seven out of 50 samples (94%) were positive to one or multiple of the studied agents. In this sense, single infection by bacterial or viral agents was found in 15 and three cases, respectively. Thirty cases of coinfection by more than one etiology were also detected, either by different bacteria (*n* = 24), which was the most frequent finding in this study, or by a combination of viruses and bacteria (*n* = 6) in the same individual (Supplementary Table S3). Bacterial infection was more prevalent in cases of both acute fibrinous (*p* < 0.001) and chronic catarrhal pneumonia (*p* < 0.01) than viral infection. *Mycoplasma bovis* (60%, 33/50) and *Mannheimia haemolytica* (40%, 20/50) were the most frequently found agents (Table 6). Viral DNA was only identified in samples showing chronic catarrhal pneumonia (Table 6), in three cases as a unique etiological agent, and in six together with bacteria. No differences in prevalence were detected between the viruses examined (Supplementary Table S4).

## 4. Discussion

BRD is considered a common disease in livestock, particularly in beef feedlots, where it has been related to poor fattening and severe economic losses [1,2,4]. This has been corroborated by the present study, where it has been shown that BRD-related pneumonic lesions have a significant prevalence (17.9% in this study) in slaughtered, clinically healthy beef cattle and are associated with a reduction in carcass weight and an increase in fattening days in the different management system studied.

Previous reports have stated that the prevalence of pneumonia varies depending on country, management system, etiological agents involved, breed or season entering the feedlot [4,5,6,7,20,21]. The findings of the present study suggest that BRD prevalence in Spain is similar to that previously described in different countries [6,7,26] and close to the data gathered in post-mortem studies in the US [19,25] and Europe [20,27]. The high rate of BRD lesions in animals with apparently healthy status at the slaughterhouse time point found in this study supports preceding publications that have pointed out that only 25% of the animals suffering severe BRD lesions would show clinical signs of the disease [2,14].

These results reflect that BRD is a multifactorial syndrome linked to the influence of several variables. With respect to age, the prevalence tends to be higher in veal calves than in yearlings. This could be associated with a diversity of factors such as their naïve immune system, frequent contact with newly introduced animals in the feedlot, prophylaxis mistakes, poor weaning period and different production systems that seem to affect managed veal calves more intensely than yearlings [14,27,28].

Some authors suggested that intensive co-housing boosts the exchange of airborne pathogens and facilitates nose-to-nose contact between calves, potentially increasing the risk of BRD [29,30]. However, in this study, when global data were considered, the highest prevalence rates of subclinical BRD were found in veal calves raised in mixed and extensive management systems. This fact could be due to some circumstances such as the mix of animals of different ages [16], the existence of less controlled environmental conditions and poor daily observation [28,31] that would occur more frequently in mixed–extensive than in intensive management systems. All these aspects deserve further investigation since, at present, the majority of studies that deal with the impact of production systems on pneumonia focus on intensive systems, and no information on the relevance of other managements on BRD is available, highlighting the significance of this work. The season of entry to feedlot and the time of birth in the extensively reared animals could be a factor to take into account that can influence the prevalence of pneumonia [32], particularly if the study were carried out at a single point. However, this study covers a long period and includes two types of animals with greater or lesser age (veals or yearlings). Thus, it does not seem likely that the season of entry to feedlot would have influenced the results since the animals included cover all times of the year.

In this study, differences were observed in the prevalence of BRD in relation to the sex of the animal on the whole. According to the results, pneumonia could be found with higher probability in males. However, when sex was analyzed together with the type of management and age, it was striking that BRD prevailed among females from the mixed and extensive systems. This could be likely related to several factors including hide thickness, fat depth, temperament, and response to stimuli that would make females prone to respiratory disease under these management circumstances [33]. In relation to the effect that the presence of lesions due to a subclinical pneumonia may have on carcass weight, the results show a decrease similar to other studies including animals with a clinical history of pneumonia [34]. According to these results, chronic catarrhal pneumonia is the most prevalent type found in slaughterhouse animals regardless of age group, gender or management systems. This was an expected finding since studied animals were fit for consumption without evident clinical signs, as occurs mostly in this type of chronic pneumonia [7,11]. Nevertheless, acute fibrinous pneumonia usually has an acute course with clinical signs such as fever or respiratory distress [12] that were not observed in the present work, probably due to the limited extent of the fibrinous pneumonic lesions found [7,11]. Among mixed or extensively managed animals, acute fibrinous pneumonia acquired more relevance, a fact that could be linked to the environmental conditions or an insufficient observation by farmers that takes place in this type of system [4,16,28].

In relation to the extent of lesions, differences were only found in animals with chronic catarrhal pneumonia, where those with grade 1 were the most frequent. This finding might be explained by the fact that a high number of cattle with this type of lesion showed changes associated with recovery as denotes the presence of areas of atelectasis, always with a low entity, affecting reduced areas of parenchyma. This could imply that chronic lesions are the consequence of events experienced in the past, mainly in calfhood [20,26], in which lesions tend to recover with tissue reorganization and are reduced in terms of extent and severity [35]. However, no differences in lesion severity were found in cases of acute fibrinous pneumonia. Lesions never affected areas greater than 30% and were unrelated to clinical signs. Thus, it would seem feasible that in some animals, always in low numbers, stress or other factors perhaps related to the management [20,26] could have complicated and exacerbated a previously existing chronic catarrhal pneumonia, leading to a more severe acute form.

One noticeable finding of this study is the demonstration of the association of pneumonic lesions with a lower carcass weight in animals with the same average days on feeding. Supporting these results, it has been reported that subclinical BRD could cause a decrease between 0.125 to 0.350 kg in the average daily gain (ADG) during the finish feeding [20,36]. In addition, animals with pneumonia arrive at the slaughterhouse with less age than the healthy ones, which would aggravate the decrease in the average weight loss. The early slaughter of these animals could be due to the decrease in ADG due to a pneumonic process and a decrease in the daily conversion rate that leads farmers to an earlier slaughter [34,36], a fact observed among intensive-production animals, where decisions are usually made based on productive parameters.

Specifically, the significant reduction of the carcass weight was observed only in intensively reared males. This could be related to the fact that extrinsic factors affecting the ADG, such as pneumonia, would have a greater impact in intensive systems where males could reach the highest productive performance [1,32,34]. Under extensive management conditions, the ADG is lower, and for pneumonia to have an effect, it should be associated with the extent of the lesion.

Indeed, when considering the macroscopic characteristics of the lesions, it was surprising to find that there were no differences in the influence of the different types of pneumonia on the carcass weight, even with those characterized by an acute course. This could be associated with the subclinical nature of all the lesions found and the limited extension of those acute cases whose clinical effect is related to the extent of pulmonary damage, and thus, cattle with mild disease would resemble the healthy cattle [37]. The pathogen profiles regarding the type of the lesion are difficult to compare, since previous studies did not consider the different types of pneumonic lesions; rather, they only took into account the existence of consolidation in the lungs [38].

It is clear that infectious agents play a key role in BRD pathogenesis [13,31], as demonstrated in this study. In Spain there are few publications regarding the etiology involved in BRD, and most of them are based on serological or swab PCR approaches. In this study, all of the pathogens screened by PCR were detected. Although PCR only indicates the presence of nucleic acids, it should be noted that samples came from affected tissue, suggesting that the pathology present was linked to the pathogen identified [39]. In the effort to avoid variability, although an intra-rater agreement calculation was not made, samples were processed in duplicate, at the same time, by a single person and with the same equipment. In case of an inconclusive result, an additional test was performed.

In the current study, at the time of slaughter, bacterial agents were more commonly found than viruses in relation to BRD lesions. Particularly, among the bacteria and in agreement with other studies [4,11,40], *Mycoplasma bovis* and *Mannheimia haemolytica* have been shown to be the most prevalent, either alone or coexisting with other bacteria or viruses. Besides, the importance of mixed infections with *Mannheimia haemolytica* and *Mycoplasma bovis*, also found by previous works, should be highlighted [18,41]. Other pathogens also reported as participating in BRD [4,17] such as *Pasteurella multocida* or *Histophilus somni,* have also been found in the current work, either alone or, more often, in coinfections with other agents. Our findings share with other studies the relevant involvement of *Mycoplasma bovis* in chronic bronchopneumonic lesions but also in acute fibrinous cases in combination with *Mannheimia haemolytica* [17,19]. Concerning viruses, finding such a low number of positive identifications, especially as unique agents was not expected, which is in line with some studies in the United States that have reported that viruses such as BRSV or BVDV are found in 14.1% of calves showing clinical respiratory disease [4,7,17] or 9% in macroscopic lesions at the point of cull [6]. The exact role of viruses on the pathogenesis of BRD is still unclear. It has been suggested that they could interfere with lung defense mechanisms and predispose calves to bacterial pneumonia [15,19]. It is then possible that viruses might have participated in the initiation of the lung lesions found in this study, but once the bacteria colonized the damaged tissue, most viruses were eliminated by the immune response of the host. The fact that viral infections could play a secondary role in BRD in this region is a possibility. This study has been conducted in the largest region of Spain with a significant beef cattle industry where, at this moment, no previous studies on the etiology of BRD have been conducted. In the present study, the only relation found between etiological agents assessed by PCR and the type of pneumonia was that acute fibrinous pneumonia was caused only by bacterial agents, mainly *Mannheimia haemolytica*, in agreement with previous reports that found this bacterium as the unique agent of this type of lesion [6]. In chronic catarrhal pneumonia cases, both bacteria and viruses were present, similar to what is described by previous studies [4,6,41]. Likewise, there was not a clear relationship between the infectious agent identified in the samples and the grade of macroscopic lesions.

## 5. Conclusions

According to these results, subclinical pneumonia is a multifactorial disease that has significant relevance in apparently healthy beef cattle submitted to the slaughterhouse, in which the prevalence and severity vary depending on the management system, age and gender. BRD causes a negative impact, in otherwise healthy animals, on production variables such as carcass weight or days of fattening, and more enhanced in those reared under intensive management systems. This study highlights the importance that through the postmortem sampling of lungs in slaughterhouses as a monitoring of the presence of the BRD, feedlots could develop and improve health programs, use of specific treatments or prophylaxis to minimize the impact of this disease and contribute significantly to the health, well-being and productivity in beef cattle herds.

## Figures and Tables

**Table 1:** Number and distribution of animals studied based on the management system, age group and gender. **♀**: female; **♂**: male.

Management System	Veals			Yearlings			Total
	♀	♂	Total	♀	♂	Total	
Intensive	509	127	636	135	136	271	907
Mixed	37	52	89	22	30	52	141
Extensive	7	21	28	9	16	25	53
	553	200	753	166	182	348	1101

**Table 2:** Parameter estimates (for an average farm) of the multilevel random intercept model predicting log odds of pneumonia (log-probability of pneumonia/probability of being healthy) and crude and conditional estimated prevalence of pneumonia.

	LS Mean (SE) ^1^	Odds Ratio (95% CI) ^2^	*p*-Value	Crude Prevalence ^3^	Adjusted Estimated Prevalence ^4^
**Management system**					
Intensive	−1.93 (0.732)	Reference		0.170	0.127
Mixed	−2.40 (0.949)	0.59 (0.03–10.79)	0.703	0.225	0.083
Extensive	−1.41 (0.986)	1.61 (0.09–27.28)	0.683	0.245	0.197
**Type of animal**					
Veal	−1.24 (0.530)	Reference		0.194	0.223
Yearling	−2.58 (0.583)	0.26 (0.13–0.54)	0.0003	0.153	0.070
Sex					
Male	−2.48 (0.559)	Reference		0.186	0.078
Female	−1.39 (0.552)	3.09 (1.54–6.19)	0.001	0.178	0.206
**System × Animal type**					
Intensive Veal	−1.79 (0.735)	Reference		0.175	0.143
Intensive Yearling	−2.07 (0.746)	0.76 (0.25–2.26)	0.204	0.159	0.111
Mixed Veal	−1.49 (0.933)	Reference		0.278	0.184
Mixed Yearling	−3.32 (1.042)	0.17 (0.05–0.48)	0.001	0.135	0.035
Extensive Veal	−0.46 (1.034)	Reference		0.357	0.388
Extensive Yearling	−2.35 (1.134)	0.15 (0.02–0.88)	0.036	0.120	0.087
**System × Sex**					
Intensive male	−1.81 (0.743)	Reference		0.202	0.141
Intensive female	−2.05 (0.736)	0.78 (2.63–20.06)	0.242	0.157	0.114
Mixed male	−3.40 (1.019)	Reference		0.134	0.032
Mixed female	−1.41 (0.947)	7.23 (0.89–29.82)	0.0001	0.350	0.196
Extensive male	−2.23 (1.044)	Reference		0.189	0.097
Extensive female	−0.59 (1.121)	5.16 (0.52–1.17)	0.067	0.375	0.358

^1^ LS: Least square means on the logit scale and standard error of the mean (SE); ^2^ CI: confidence limits; ^3^ Number of pneumonia cases/total animal; ^4^ Least square means on the probability scale (LS means were adjusted for an average farm).

**Table 3:** Parameter estimates of the model predicting log odds of type of pneumonia (log-probability of acute fibrinous /probability of chronic pneumonia) and crude observed and estimated prevalence of acute fibrinous pneumonia.

	LS mean (SE) ^1^	Odds Ratio (95% CI) ^2^	*p*-Value	Crude Prevalence ^3^	Estimated Prevalence ^4^
**Management system**					
Intensive	−2.22 (0.292)	Reference		0.091	0.098
Mixed	−1.17 (0.448)	2.86 (1.03–7.915)	0.043	0.219	0.236
Extensive	−0.47 (0.594)	5.77 (1.61–20.642)	0.007	0.385	0.384
**Type of animal**					
Veal	−1.23 (0.448)	Reference		0.123	0.206
Yearling	−1.35 (0.298)	1.12 (0.41–3.07)	0.820	0.151	0.226
**Sex**					
Male	−1.00 (0.344)	Reference		0.268	0.268
Female	−1.58 (0.386)	0.56 (0.22–1.42)	0.224	0.171	0.171

^1^ LS: Least square means on the logit scale and standard error of the mean (SE); ^2^ CI: confidence limits; ^3^ number of fibrinous pneumonia cases/total cases of pneumonia; ^4^ Least square means on the probability scale (farm nested to system was not included in the model because of its variance was zero).

**Table 4:** Effect of the subclinical pneumonia on carcass weight (kg) at slaughter for the different production systems (IM: intensive management; MM: mixed management; EM: extensive management), type of animal (Veal vs. Yearling) and sex.

	Production System			Type of Animal		Sex		*p*-Values ^1,2^			
	IM	MM	EM	Veal	Yearling	♀	♂	*p*	*p* × Sys	*p* × Type	*p* × Sex
No lesions	273 ^b^	266	258	234 ^c^	297	234	297 ^b^	0.772	0.082	0.214	0.0006
Pneumonia	256 ^a^	268	262	225 ^d^	299	244	280 ^a^				
SED ^3^	4.4	9.9	15.9	6.8	8.6	7.9	7.4				

^1^*p*: Probability value for the effect of health status; *p* × Sys: probability value for the effect of health status × production system interaction; *p* × Type: probability value for health status × type of animal interaction; *p* × Sex: probability value for health status × sex of animal interaction; ^2^ other effects: system (*p* = 0.8390); type of animal (*p* < 0.0001); sex (*p* < 0.0001), triple interaction were not significant (*p* > 0.05); ^3^ SED: standard error of the difference; ^a,b^ means with different superscript within the same column are significantly different (*p* < 0.05); ^c,d^ means with different superscript within the same column tend to be significantly different (*p* < 0.15). ♀: female; ♂: male.

**Table 5:** Effect of the subclinical pneumonia on age at slaughter (months) at slaughter for the different production (IM: intensive management; MM: mixed management; EM: extensive management), type of animal (Veal vs. Yearling) and sex.

	Production System			Type of Animal		Sex		*p*-Values ^1,2^			
	IM	MM	EM	Veal	Yearling	♀	♂	*p*	*p* × Sys	*p* × Type	*p* × Sex
No lesions	12.7 ^b^	12.7 ^c^	12.5	10.0	15.2	12.3	13.0 ^d^	0.878	0.042	0.964	0.005
Pneumonia	12.3 ^a^	13.4 ^d^	12.0	10.0	15.2	12.7	12.4 ^c^				
SED ^3^	0.19	0.38	0.63	0.28	0.36	0.32	0.31				

^1^*p*: probability value for the effect of health status; *p* × Sys: probability value for the effect of health status × production system interaction; *p* × Type: probability value for health status × type of animal interaction; *p* × Sex: probability value for health status × sex of animal interaction; ^2^ other effects: system (*p* = 0.0837); type of animal (*p* < 0.0001); sex (*p* = 0.2826), triple interaction were not significant (*p* > 0.05); ^3^ SED: standard error of the difference; ^a,b^ means with different superscript within the same column are significantly different (*p* < 0.05); ^c,d^ means with different superscript within the same column tend to be significantly different (*p* < 0.10). ♀: female; ♂: male.

**Table 6:** Summary of the different etiologies found in each type of lesion by polymerase chain reaction (PCR) *n*: number.

Etiological Diagnosis Real-Time PCR (RT-PCR)	Chronic % (*n* = 40)	Acute % (*n* = 10)	Total % (*n* = 50)
*Mannheimia haemolytica*	37.5% (15/40)	50% (5/10)	40% (20/50)
*Mycoplasma bovis*	65% (26/40)	70% (7/10)	60% (33/50)
*Histophilus somni*	15% (6/40)	20% (2/10)	16% (8/50)
*Pasteurella multocida*	27.5% (11/40)	10% (1/10)	24% (12/50)
Bovine Herpesvirus type I	5% (2/40)	0	4% (2/50)
Bovine Respiratory sincitial virus	10% (4/40)	0	8% (4/50)
Parainfluenza virus type 3	10% (4/40)	0	8% (4/50)
Bovine Viral Diarrhea virus	2.5% (1/40)	0	2% (1/50)

## Supplementary Materials

The following are available online at https://www.mdpi.com/2076-2615/10/10/1770/s1, Table S1: Commercial kits employed to perform RT-PCR analysis, Table S2. Prevalence of pneumonia in relation with age and management system, Table S3. Parameter estimates of the model predicting log odds of severity of lesions (log-probability of be grade II (severe)/probability of be grade I (mild)) and crude and estimated occurrence of severe lesion, Table S4: Etiological agents identified by PCR in each type of pneumonia.
