# Supplementary material for: Production Significance of Bovine Respiratory Disease Lesions in Slaughtered Beef Cattle
Source: Animals (Basel). 2020 Sep 30;10(10):1770. doi: 10.3390/ani10101770 (PMC7599887; doi:10.3390/ani10101770)
Supplement: Supplementary file 1 [file animals-10-01770-s001.pdf]

Table S1. Commercial kits employed to perform RT-PCR analysis. Positive results were assumed when an amplification curve was observed before cycle 45 (Ct < 45).

Etiology	Commercial kit
Mycoplasma bovis	LSI VetMAX™ *M. bovis* Kit, Applied Biosystems®, US
Histophilus somni	LSI VetMAX™ *H. somni* Kit, Applied Biosystems®, US
Mannheimia haemolytica	BactoReal® Kit *M.haemolytica*, Ingenetix®, Austria
Pasteurella multocida	LSI VetMAX™ *P. multocida*, Applied Biosystems®, US
BVDV	LSI VetMAX™ *BVDv* Screening, Applied Biosystems®, US
BHV-1	BoHV-1 dtec qPCR Test, Genetic PCR Solutions®, Spain
BRSV and PI-3	LSI VetMAX™ Triplex bRSV & PI-3 kit, Applied Biosystems®, US

Table S2. Prevalence of pneumonia in relation with age and management system. IM: intensive management; MM: mixed management; EM: extensive management; n: number.

**Management system**	**Total % (n)**	**Veal**	**Yearling**
**Intensive**	16.86 % (153/907)	17.45 % (111/636)	15.87 % (43/271)
**Mixed**	21.98 % (31/141)	26.97 % (24/89)	13.46 % (7/52)[b]
**Extensive**	24.53 % (13/53)	35.71 % (10/28)	12.00 % (3/25)
**Global (IM+ MM + EM)**	17.98 % (198/1101)	19.26 % (145/753)	15.23 % (53/348)

Table S3. Parameter estimates of the model predicting log odds of severity of lesions (log -probability of be grade II (severe)/probability of be grade I (mild)) and crude and estimated occurrence of severe lesion.

	LS mean (SE)[1]	Odds ratio (95% CI)[2]	P- value	Crude prevalence[3]	Estimated prevalence[4]
**Type of pneumonia**					
Chronic	-0.56 (0.278)	Reference		0.405	0.363
Acute	-0.28 (0.426)	1.33 (0.546-3.246)	0.5270	0.461	0.431
**Production system**					
Intensive	-0.21 (0.255)	Reference		0.435	0.449
Mixed	-1.12 (0.438)	0.40 (0.167-0.973)	0.0434	0.250	0.247
Extensive	-0.06 (0.574)	1.31 (0.399-4.276)	0.6568	0.538	0.515
**Type of animal**					
Veal	-0.20 (0.284)	Reference		0.432	0.451
Yearling	-0.64 (0.375)	0.64 (0.316-1.302)	0.2171	0.358	0.345
**Sex**					
Male	-0.29 (0.317)	Reference		0.437	0.427
Female	-0.55 (0.332)	0.78 (0.406-1.484)	0.4420	0.398	0.367

[1] LS: Least square means on the logit scale and standard error of the mean (SE); [2] CI: confidence limits; [3] Number of cases with severe lesions/total of pneumonia cases; [4] Least square means on the probability scale (farm nested to system was not included in the model because of its variance was not different from zero).

Table S4. Etiological agents identified by PCR in each type of pneumonia. M. hae: *Mannheimia haemolytica*; M. bov: *Mycoplasma bovis*; H. som: *Histophilus somni*; P. mult: *Pasteurella multocida*; BHV-1: bovine herpesvirus serotype 1; BRSV: bovine respiratory syncytial virus; PI-3: parainfluenza virus type 3; BVDV; bovine viral diarrhea virus.

**Etiological diagnosis RT-PCR**	**Chronic (40)**	**Acute (10)**	**Total (n=50)**
M. hae	2	1	3
M. bov	8	2	10
H. som	2	0	2
P. mult	1	0	1
M.hae + M.bov	6	3	9
M.hae + M.bov + H.som	0	1	1
M.hae + M.bov + P.mult	2	0	2
M.hae + P.mult	1	0	1
M.hae + M.bov + H.som + P.mult	1	0	1
M.bov + H.som	3	1	4
M.bov + P.mult	3	1	4
BHV-1	1	0	1
BRSV	1	0	1
PI-3	1	0	1
BHV-1 + M.hae + M.bov	1	0	1
BRSV + P.mult	1	0	1
BRSV + PI-3 + M.hae + M.bov	2	0	2
PI-3 + P.mult	1	0	1
BVDV + P.mult	1	0	1
